# miR-135b suppresses tumorigenesis in glioblastoma stem-like cells impairing proliferation, migration and self-renewal

**DOI:** 10.18632/oncotarget.5925

**Published:** 2015-09-30

**Authors:** Valentina Lulli, Mariachiara Buccarelli, Maurizio Martini, Michele Signore, Mauro Biffoni, Stefano Giannetti, Liliana Morgante, Giovanna Marziali, Ramona Ilari, Alfredo Pagliuca, Luigi Maria Larocca, Ruggero De Maria, Roberto Pallini, Lucia Ricci-Vitiani

**Affiliations:** ^1^ Department of Hematology, Oncology and Molecular Medicine, Istituto Superiore di Sanità, Rome, Italy; ^2^ Institute of Anatomic Pathology, Università Cattolica del Sacro Cuore, Rome, Italy; ^3^ Institute of Human Anatomy, Università Cattolica del Sacro Cuore, Rome, Italy; ^4^ Regina Elena National Cancer Institute, Rome, Italy; ^5^ Institute of Neurosurgery, Università Cattolica del Sacro Cuore, Rome, Italy

**Keywords:** ADAM12, SMAD5, GSK3β, miRNAs, glioblastoma, glioblastoma stem cells

## Abstract

Glioblastoma multiforme (GBM) is the most common and fatal malignant adult primary brain tumor. Currently, the overall prognosis for GBM patients remains poor despite advances in neurosurgery and adjuvant treatments. MicroRNAs (miRNAs) contribute to the pathogenesis of various types of tumor, including GBM. In this study we analyzed the expression of a panel of miRNAs, which are known to be differentially expressed by the brain and GBM tumor, in a collection of patient-derived GBM stem-like cells (GSCs). Notably, the average expression level of miR-135b, was the most downregulated compared to its normal counterpart, suggesting a potential role as anti-oncogene.

Restoration of miR-135b in GSCs significantly decreased proliferation, migration and clonogenic abilities. More importantly, miR-135b restoration was able to significantly reduce brain infiltration in mouse models of GBM obtained by intracerebral injection of GSC lines. We identified ADAM12 and confirmed SMAD5 and GSK3β as miR-135b targets and potential mediators of its effects. The whole transcriptome analysis ascertained that the expression of miR-135b downmodulated additional genes driving key pathways in GBM survival and infiltration capabilities.

Our results identify a critical role of miR-135b in the regulation of GBM development, suggesting that miR-135b might act as a tumor-suppressor factor and thus providing a potential candidate for the treatment of GBM patients.

## INTRODUCTION

Glioblastoma (GBM) is the most common and fatal malignant primary brain tumor in adults. Despite advances in treatment strategies combining surgery, radiotherapy and chemotherapy, the prognosis of patients remains poor, with a median survival time of approximately 12 to 14 months. The extremely poor prognosis of GBM is largely due to the high tendency of invasion of the surrounding brain, incomplete surgical resection, and high frequency of tumor recurrence [1].

Growing evidence has led to the identification of a sub-population of tumor cells with stem-like properties, glioblastoma stem-like cells (GSCs), which specifically has a high capacity to resist or to adapt to standard therapies, leading to therapeutic resistance [2]. Thus, the development of efficient therapies that are able to target these cells are urgently needed. The molecular mechanisms underlying the aggressive malignant phenotype of high grade glioma cells, are still largely unknown. However, the molecular characterization of GBM is unveiling new potential therapeutic strategies based on targeting specific components of oncogenic pathways, e.g., by delivering a therapeutic gene or microRNA (miRNA) [3-6]. Several miRNAs are differentially expressed in a variety of malignancies compared to corresponding healthy tissues. Some of these miRNAs have been shown to play important roles in cancer cell proliferation, aggressiveness and metastasis. Therefore, miRNAs might hold a great potential in the future treatment of GBM tumor. This prompted us to identify and to study miRNAs deregulated in GSCs isolated from GBM patients.

In this report, we describe miR-135b as a miRNA that is significantly downregulated in GSCs compared to normal adult neural stem cells. The first evidence of a correlation between miR-135b and cancer emerged from a global miRNA expression profiling of microdissected tissues from pancreatic ductal adenocarcinoma (PDAC) patients that identified miR-135b as a novel biomarker for this tumor type [7]. Further studies revealed that dysregulation of miR-135b has a critical role in cancer progression. Particularly, the overexpression of miR-135b has been described in human head and neck squamous cell carcinoma (HNSCC) cell lines, where it causes increased cell proliferation, migration, and colony formation [8]; in human breast cancers, where it correlates with patient survival and early metastatization [9]; in highly invasive non-small-cell lung cancer cells where promotes cancer metastasis [10]. Moreover, miR-135b has been proposed as a biomarker and potential therapeutic target for colorectal cancer (CRC) and advanced adenoma [11]. Indeed, upregulation of this miRNA is common in sporadic and inflammatory bowel disease-associated human CRCs and correlates with tumor stage and poor clinical outcome [11]. miR-135b is frequently amplified and upregulated in hepatocellular carcinoma (HCC) and promotes HCC invasion and metastasis [12]. More recently, it has been demonstrated that miR-135b expression is frequently upregulated in osteosarcoma and promotes cell proliferation, migration and invasion [13]. In brain tumors miR-135b expression has been shown to be significantly higher in GBM compared to lower grade gliomas [14] as well as in frankly tumoral compared to peritumoral areas of GBM samples [15]. Altogether, these data strongly suggest that miR-135b behaves as an onco-miRNA promoting tumor cells proliferation and invasion or as a key downstream effector of oncogenic pathways promoting tumor transformation and progression. In contrast to this view, we here describe that miR-135b is highly downregulated in our GSC line collection compared to their normal neural stem cells. More importantly, forced expression of miR-135b into GSCs markedly suppressed proliferation, motility and invasion of glioma cells as well as their stem cell-like phenotype through targeting ADAM12, SMAD5 and GSK3β. Furthermore, our data show that miR-135b inhibits glioma tumorigenesis and invasion in the brain of mice. This led us to assess the overall transcriptome changes upon enforced miR-135b expression in GSC lines, observing the modulation of transcripts belonging to additional pathways that play a relevant role in tumorigenesis.

## RESULTS

### miR-135b expression is decreased in human GSC lines

In order to identify miRNAs relevant for GSC tumorigenesis, we analyzed by real time PCR a panel of 48 miRNAs previously described as differentially expressed between normal and tumor tissue in GBM patients [16-21], in a collection of GSC lines [22] compared with normal neural stem cell lines isolated from human adult olfactory bulb and foetal brain [23]. Among the miRNAs analyzed, 18 were detectable and dysregulated with at least a twofold variation compared to normal neural stem cells. The expression trend of 12 of them (i.e. miR-21, miR-10b and miR-124) was in line with previously published results, whereas 2 miRNAs (miR-137 and miR-218) showed an opposite trend [24]. The remaining 4 miRNAs were invaluable due to insufficient or conflicting literature data.

**Figure 1:**
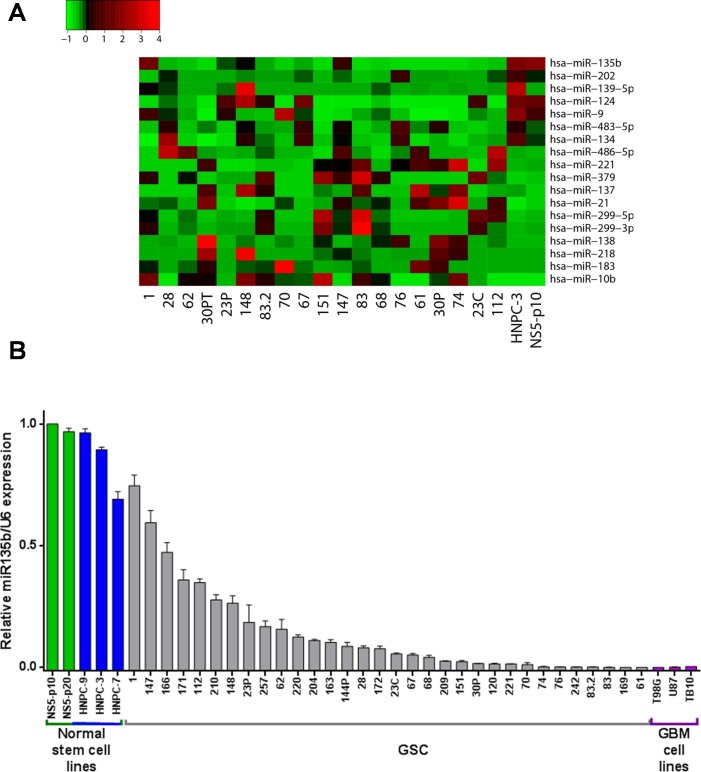
miR-135b is downregulated in GSC lines **A.** Cell plot of expression values for several miRNAs (rows) in a cohort of GSCs and in 2 neural stem cell lines (columns, numbered). Ct values from real-time PCR were converted into relative abundance values and the scale ranges from low (green) to high (red). **B.** Expression of miR-135b detected by real-time PCR in a large collection of GSCs (grey), human neural adult (green) and fetal (blue) stem cells and GBM cell lines (purple). Relative expression values represent mean and SD from three independent experiments.

miR-135b showed the most homogeneous profile in the GSC lines analyzed among the most downregulated miRNAs (Figure 1A). Being this observation apparently in contradiction with those published so far, we verified miR-135b expression in a larger panel of GSCs, as well as in the primary TB10, and commercially available T98G and U87MG, GBM cell lines (Figure 1B). Furthermore, a low expression of miR-135b was confirmed in a panel of 16 GBM samples, 11 of which derived from patients that gave raise to GSC cultures (Supplementary Figure S1a, left panel). Compared to 4 normal brain tissues, derived from patient deceased for non oncological causes, GBM samples showed a slight but not significant increase of miR-135b expression, whereas GSCs, even though with a less homogeneous distribution, displayed a higher expression of miR-135b. Both GSCs and GBM samples, expressed lower level of miR-135b compared to normal neural stem cell reference. Interestingly, a similar behavior was observed for miR-21 (Supplementary Figure S1a, right panel), known to be upregulated in many tumors including GBM [17]. This observation may suggest that microenvironment may regulate miRNAs expression. Thus, to evaluate micro environmental effects on miR-135b expression mouse xenogenic tumor by intracranial and subcutaneous injection of GSCs were generated (Supplementary Figure S1b). Decreased miR-135b and miR-21 levels were observed in both xenograft models as compared to cultured tumor generating GSCs, indicating that microenvironment may contribute to miRNA regulation. However, we cannot exclude that cellular heterogenic composition of tumor tissue could account for the observed decrease of miR-135b and miR-21 levels in both xenograft models compared to cultured tumor generating GSCs.

To further confirm the data on miR-135b expression, we analyzed by *In Situ* Hybridization (ISH) a panel of 12 GBM specimens and 4 normal brains derived from patients deceased for non oncological causes (Supplementary Figure S1c). Both patients and normal brains showed a highly heterogeneity of miR-135b expression ranging from complete negativity (Supplementary Figure S1c, panel D), few scattered positive cells (panel E) to more abundant positivity (panel C and F) with no significant differences between normal and GBM samples. Moreover, miR-135b expression in GBM samples was markedly lower than in colon adenocarcinoma used as the positive control (panel A).

### Restoration of miR-135b impairs tumorigenic properties of GSCs *in vitro*

**Figure 2:**
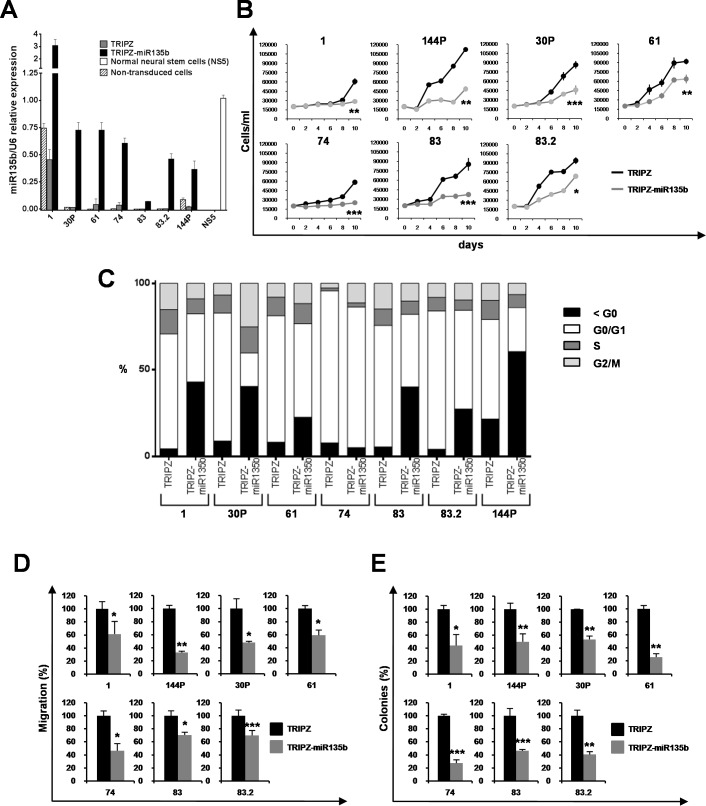
miR-135b overexpression reduces cell growth, migration and clonogenic abilities of GSCs **A.** Real-time PCR levels of miR-135b in GSCs non-transduced and transduced with either TRIPZ or TRIPZ-miR-135b inducible vectors after doxycycline exposure and in NS5 normal neural stem cell line. Values are mean and SD from at least three independent experiments in duplicate. **B.** Growth curves of individual GSCs (line number on top of each plot) transduced with either TRIPZ or TRIPZ-miR-135b vectors. Points and range lines at each day represent mean and SD of at least two independent experiments in triplicate. Two-way analysis of variance for repeated measures, performed on the whole set of data demonstrated a significant growth reduction by miR-135b in all GSCs (*p* = 0.000167). **C.** Cell cycle phase distribution in TRIPZ and TRIPZ-miR-135b GSCs 6 days after induction. Representative values from three independent experiments are shown for each GSC line and are compared to the correspondent empty vector control. **D.** Analysis of migration efficiency in GSCs transduced with miR-135b 48h after induction. Values are reported as per cent relative to control vector and shown as mean ± SD from two independent experiments in duplicate. Analysis of variance demonstrated a significant effect of miR-135b restoration on the ability of GSCs to migrate (*p* < 0.0001). **E.** Analysis of efficiency in colony formation of GSCs after transduction with TRIPZ-miR-135b. Percent colony number values from two independent experiments in duplicate were calculated over the correspondent empty vector and are shown as mean ± SD for each GSC line. Analysis of variance demonstrated a significant effect of the presence of miR-135b on the colony-forming ability of GSCs (*p* < 0.0001).

To further investigate the role of miR-135b on GCS tumorigenic properties we over-expressed miR-135b in GSC lines by using an inducible Tet-On lentiviral vector (TRIPZ) carrying pri-miR-135b in the 3′ untranslated region of Red Fluorescent Protein (RFP). Seven GSC lines (#1, #30P, #61, #74, #83, #83.2 and #144P) chosen as representatives of different levels of miR-135b expression, were transduced and exposed to doxycycline. RFP-positive cells were flow-sorted and miR-135b restoration was confirmed by real-time PCR (Figure 2A). All GSC lines transduced with this vector showed miR-135b expression levels comparable to normal cells or increased when compared to control vector (TRIPZ)-transduced cells.

Ectopic expression of miR-135b significantly impaired cell growth of all the GSC lines tested (Figure 2B), with a stable decrease in growth rate. To better characterized the inhibitory effect of miR-135b restoration on cell proliferation, we performed BrdU incorporation assay on 2 out of 7 cell lines, GSC #83, chosen as it shows the lowest ectopic expression of miR-135b, and GSC #144P, representative of the average levels achieved after transduction (Figure 2A). miR-135b significantly reduced BrdU incorporation in both cell lines independently of the levels of endogenous and restored miRNA expression (Supplementary Figure S2a) indicating a decreased progression in the cell cycle through the S phase.

We further evaluated DNA cell content in TRIPZ-miR-135b- or empty vector- transduced GSCs (Figure 2C) and found a consistent increase in the pre-G_0_ peak in most miR-135b-restored GSCs. Interestingly, in GSC #74, differently from the other transduced GSC lines, no increase of pre-G_0_ peak was found and a block in G_2_/M phase was observed, suggesting that miR-135b could inhibits tumor cell proliferation through different mechanisms.

To deeply analyze the effect of miR-135b restoration on the induction of cell death we performed caspase 3/7 activity evaluation on GSC line #83 and #144P. No significant induction of apoptosis was observed in both GSC lines (Supplementary Figure S2b) suggesting that overexpression of miR-135b is able to impairs GSC cell growth mainly by inhibiting cell proliferation rather than increasing apoptosis.

We then examined whether miR-135b could alter additional malignant features of GSCs, such as migration. The motility of GSCs after miR-135b induction was examined and a dramatic reduction in the migration capabilities of TRIPZ-miR-135b GSCs (Figure 2D and Supplementary Figure S3), was observed.

Moreover, we analyzed the clonogenic capability of GSC expressing miR-135b. Doxycycline-induced cells were plated as single cells in 96 well plates in duplicate and allowed to grow for two weeks. TRIPZ-miR-135b GSCs formed significantly fewer colonies as compared to TRIPZ cells (Figure 2E). Thus, miR-135b restoration resulted in a considerable inhibition of proliferation, migration and colony formation of all the GSCs tested, suggesting that this miRNA could play a pivotal role in GBM oncosuppression.

To complement this set of experiments, endogenous miR-135b was inhibited in normal neural adult stem cells by transducing lentiviral vector carrying anti-sense miR-135b or anti-sense control sequence and green fluorescent protein (GFP) as a reporter. After transduction a 40% decrease of the endogenous levels of miR-135b was observed in GFP-positive cells (Supplementary Figure S4a). miR-135b knockdown induced only minimal variations in either estimate stem cell frequency (32.4 of anti-miR-135b *vs* 45.3 of NTC, *p* = 0.317), or BrdU incorporation (Supplementary Figure S4b) or migration (Supplementary Figure S4c). These results may be consistent with the relative small changes of miR-135b after knockdown of endogenous miR-135b.

### miR-135b restoration significantly decreases *in vivo* tumor growth

**Figure 3:**
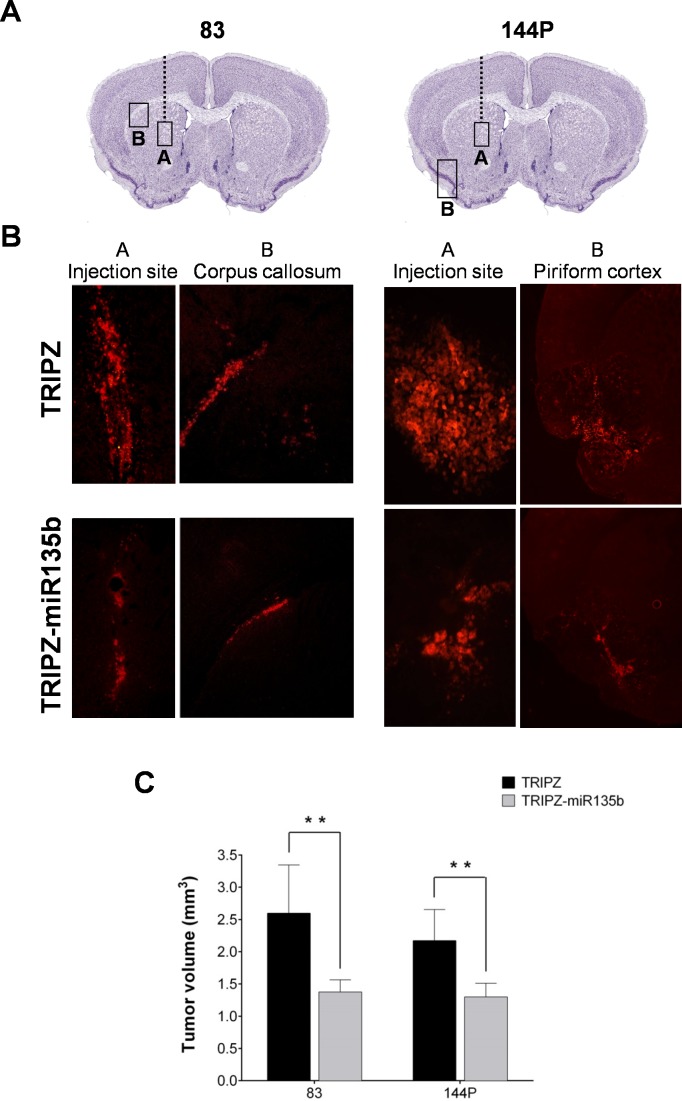
miR-135b overexpression reduces the *in vivo* growth of GSC-derived brain tumor **A.** Representative coronal sections of the mouse brain illustrating the sites of cell injection (box A) and of brain invasion (box B). Mice were injected with two different GSC lines (#83 and #144P) transduced with either empty vector or miR-135b. **B.** Fluorescence microscopy images of mouse brain specimens at eight weeks after injection of GSC #83 (left panel; A, injection site; B, corpus callosum) and of GSC #144P (right panel; A, injection site; B, piriform cortex). **C.** Analysis of the extent of tumor cell mass in the full series of brain sections from mice injected with control and miR-135b-overexpressing GSCs.

To evaluate whether our findings were confirmed *in vivo*, the TRIPZ and the TRIPZ-miR-135b GSC lines #83 and #144P were grafted into the striatum of NOD/SCID mice (Figure 3A).

Tumor growth was assessed at 8 weeks after grafting during which mice received doxycycline in drinking water. Fluorescence microscopy analysis of serial coronal brain sections showed that the degree of brain invasion was significantly reduced in TRIPZ-miR-135b GSC brain xenografts (Figure 3B). Eight weeks after grafting, control TRIPZ grafted mice (*n* = 6) harbored tumors that invaded the homolateral striatum, piriform cortex, corpus callosum, anterior commissure, internal capsule, and fimbria-hippocampus, whereas the degree of brain invasion was significantly reduced in TRIPZ-miR-135b grafted mice (*n* = 6) (Figure 3C). In mice injected with GSC #83, the volume of the brain region invaded by the red fluorescent tumor cells was 2.597 ± 0.365 and 1.376 ± 0.187 mm^3^ (mean ± sem) in TRIPZ and TRIPZ-miR-135b grafted mice, respectively (*p* = 0.041). A similar pattern was found in GSC #144P xenografts with invasion volumes of 2.172 ± 0.235 and 1.301 ± 0.194 mm^3^ (mean ± sem) in TRIPZ and TRIPZ-miR-135b grafted mice, respectively (*p* = 0.046) (Figure 3C).

In order to better characterize the effect of miR-135b restoration on tumor growth *in vivo*, we decided to set up two additional models by intracerebral injection of U87MG GBM cells and by subcutaneous injection of T98G GBM cells. As shown in Figure 1B, these cell lines express very low levels of miR-135b compared to normal neural stem-like cells, consistent with the expression in the majority of GSC lines. TRIPZ and the TRIPZ-miR-135b U87MG GBM line were grafted into the striatum of NOD/SCID mice. Twelve days after grafting, the brains injected with control U87MG cells (*n* = 5) showed the tumor growing along the needle tract from the cortex to the striatum and exerting a mass effect (Supplementary Figure S5, upper panel). In addition, spheroid aggregates of tumor cells were found in the ventricles, as a result of spreading along the cerebrospinal fluid paths. The tumor xenografts had intense proliferating activity, as assessed by DAPI staining and Ki67 immunoreaction, with mitotic index of 4.57 + 0.68 per cent (mean + SD). Conversely, the brains grafted with TRIPZ-miR-135b U87MG cells (*n* = 4) showed groups of fluorescent cells in the injected area that did not produced any mass effect on the surrounding brain parenchyma (Supplementary Figure S5, lower panel) and with no evidence of proliferating activity. Moreover, TUNEL assay did not show an increase of cell death in TRIPZ-miR-135b xenografts (data not shown) confirming that, as assessed *in vitro,* miR-135b exerts its function mainly by inhibiting proliferation than by inducing apoptosis.

To confirm the effect of miR-135b restoration in tumor growth we chose subcutaneous grafting of GSCs as Matrigel implants in immunodeficient mice, a well suited model to study the early stages of *in vivo* tumor growth [25]. Histological examination showed that four weeks after grafting the implants (*n* = 3) were populated by cluster of tumor cells and that cell proliferation was lowered in TRIPZ-miR-135b T98G xenografts compared with paired TRIPZ T98G xenografts as assessed by immunostaining with anti-Ki67 (Supplementary Figure S6).

### Tumor-suppressor function of miR-135b involved ADAM12 and SMAD5 signaling

**Figure 4:**
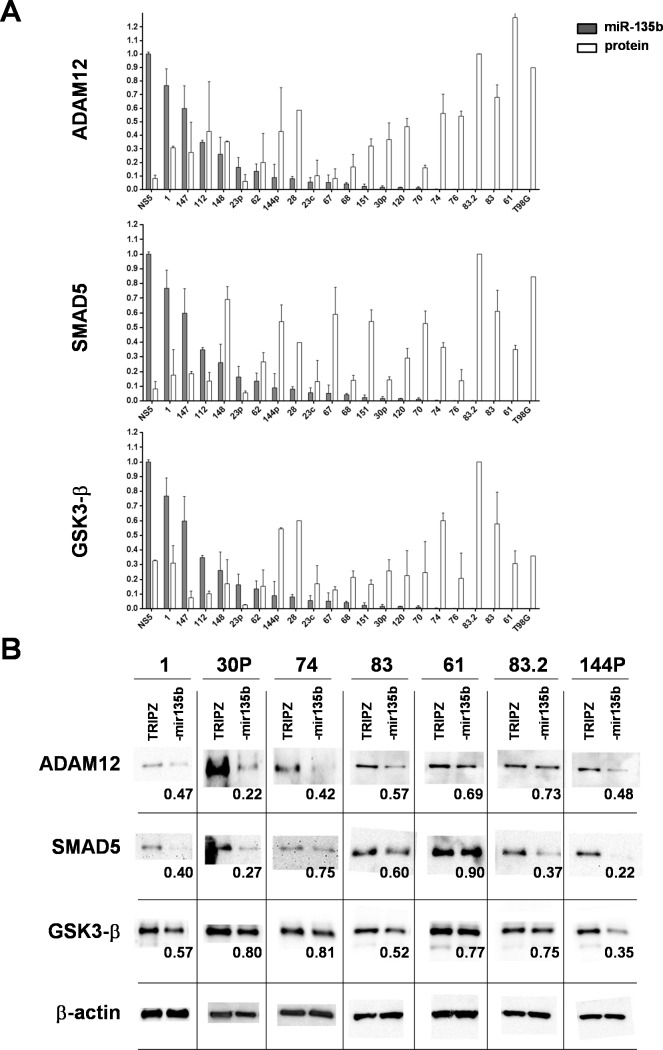
miR-135b overexpression induces a significant decrease in the level of target proteins **A.** Normalized levels of miR-135b/U6 expression were plotted with protein levels of the three main miR-135b targets in a large cohort of GSCs, T98G GBM cell line and a representative normal neural stem cell line. Protein levels, normalized with β-actin, were determined relative to GSC #83.2 showing the higher content of the three protein targets. Bars indicate the mean values ± SD of two independent experiments and show a significant inverse correlation among miR-135b and putative target levels (ADAM12 ρ = −0.635, *p* = 0.001; SMAD5 ρ = −0.484, *p*=0.022; GSK3β ρ = −0.447, *p* = 0.037). **B.** Representative western blot analyses of the three main miR-135b targets in a cohort of GSCs transduced with either TRIPZ vector or TRIPZ-miR-135b. Values below the miR-135b lanes indicate the fold change in protein levels relative to the TRIPZ control vector.

To further understand the molecular mechanism by which miR-135b can behave as tumor-suppressor, we tried to establish whether any of its putative targets might play a significant role in GBM biology. Most of the target genes identified by several target prediction engines shared a tumor-suppressor function compatible with the upregulation of this miRNA in most of the different cancers analyzed in previous studies. Since recent evidence supports the notion that miRNAs act on their target gene repertoire also at the transcriptional level affecting the transcript stability [26], we characterized at the transcriptome level the effects of miR-135b restoration in the context of GSC cell lines. To this end, we performed microarray analysis of RNA from TRIPZ and TRIPZ-miR-135b GSC cell line #83. We expected higher effect of miR-135b restoration in this GSC line since it has the lowest expression of miR-135b. We hence examined the annotation for the most downregulated genes. A list of potential target mRNAs was obtained for miR-135b using TargetScan 6.2 algorithm (www.targetscan.org). Among these, the metalloproteinase ADAM12 is highly expressed in human GBM and might play a role in the prominent proliferation of tumor cells through shedding of heparin-binding epidermal growth factor (HB-EGF) [27]. Another potential mediator of the tumor-suppressive ability of miR-135b is SMAD5, which has been recently identified as one of the target of miR-135b activated by the transcription factor PAX6 [28]. Moreover, a third miR-135b target in GSCs could be GSK3β, whose downregulation has been involved in radioresistance acquisition of a U87MG GBM cell line derivative [29]. The comparison in GSCs between the expression of miR-135b and the protein level of the three targets showed an inverse correlation (Figure 4A and Supplementary Figure S7), thus supporting the hypothesis that ADAM12, SMAD5 and GSK3β are indeed miR-135b targets in GSCs. To corroborate these results, we evaluated the expression of the three target proteins in TRIPZ and TRIPZ-miR-135b GSCs. We chose to assay the target protein levels two days after attaining full miRNA induction, in order to allow protein and transcript turnover to be affected by post-transcriptional repression. Results in Figure 4B show that ADAM12, SMAD5 and GSK3β target proteins are considerably diminished in cells after miR-135b is induced, albeit at different extent that might arise from differential affinity of the miRNA for the transcript, differential transcript/protein half-life or other factors.

To assess whether ADAM12 is a direct target of miR-135b, we cloned part of ADAM12 3′UTR into a luciferase reporter vector. Co-transfection in 293T, along with miR-135b mimic-oligonucleotide, significantly reduced the luciferase activity compared to a control oligonucleotide (scramble) (Supplementary Figure S8). The luciferase activity was restored in a construct bearing five mutations in the putative miR-135b target sequence (Supplementary Figure S8). These results strongly support a direct targeting of ADAM12 by miR-135b as previously demonstrated for the other two targets SMAD5 and GSK3β [28, 29].

### Phenotype rescue is partially established through ADAM12 re-expression

**Figure 5:**
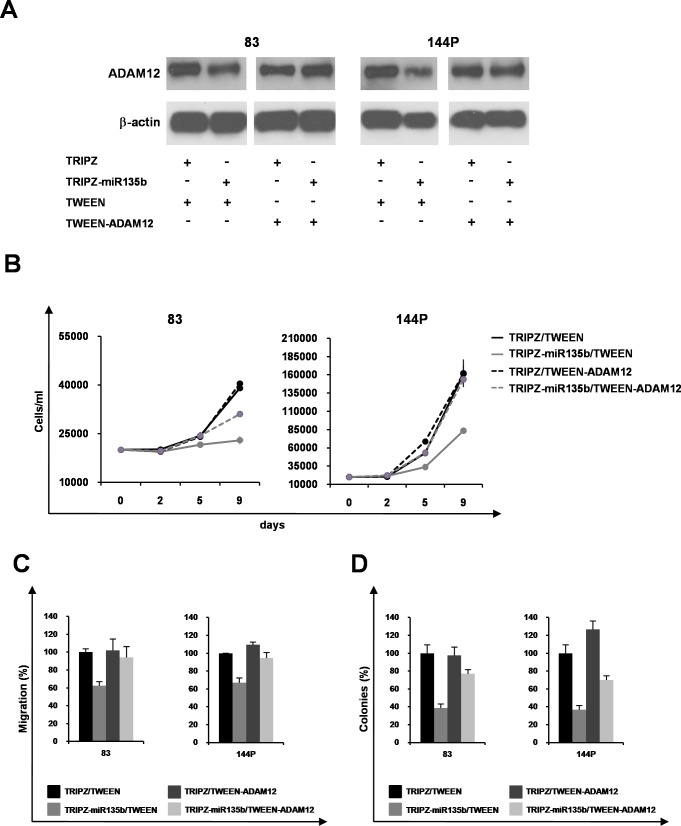
ADAM12 re-expression in GSCs partly induces phenotypic rescue **A.** Western blot analysis of ADAM12 on GSC lines #83 and #144P (TRIPZ and TRIPZ-miR-135b) transduced with either empty Tween vector or Tween-ADAM12. **B.** Growth curve of GSC lines #83 and #144P (TRIPZ and TRIPZ-miR-135b) expressing ADAM12 gene. Points and range lines at each day represent mean and SD of at least two independent experiments in triplicate. **C.** Percentage of migrating cells in GSC (TRIPZ and TRIPZ-miR-135b) after transduction with either Tween or Tween-ADAM12 vectors. Per cent values are reported as mean ± SD from two independent experiments in duplicate. **D.** Analysis of efficiency in colony formation of GSC (TRIPZ and TRIPZ-miR-135b) after transduction with Tween or Tween-ADAM12 vectors. Data are expressed as means ± SD of two independent experiments performed in duplicate.

ADAM12 is selectively expressed in GBM tissues [27] where it might be involved in supporting tumor cell proliferation that is mainly sustained by enhanced EGF signaling through EGFR. Heparin-binding EGF-like growth factor (HBEGF), a known EGFR ligand produced as a transmembrane protein needing enzymatic cleavage to be active, is an ADAM12 substrate, and has been implicated in EGFR dysregulation [30]. To ascertain whether a decrease in ADAM12 mediates the oncosuppressor properties of miR-135b and to verify phenotype recovery, functional experiments were performed. TRIPZ- and TRIPZ-miR135b GSC #83 and #144P were transduced with lentiviral constructs to restore ADAM12 target gene. Overexpression of ADAM 12 was verified by immunoblotting (Figure 5A). The results shown in Figure 5B-5D support the hypothesis that ADAM12 contributes to the oncosuppressive properties of miR-135b, as its re-expression mitigates the decreased proliferation (Figure 5B), migration (Figure 5C) and clonogenic ability (Figure 5D) imparted by this miRNA.

### Transcriptome changes associated with miR-135b expression

To gain further insight into the regulatory program of miR-135b in GSCs, we assessed the transcriptome variation upon miR-135b doxycycline-mediated induction. Microarray profiling allowed to find additional genes in the TGFβ pathway that are downmodulated in miR-135b expressing cells, such as SMAD2 and TGFBR2 (Supplementary Figure S9). A search for enrichment of modulated transcript in the KEGG pathways identified the cell adhesion molecules as the most statistically significant category to be modulated, being the only pathway enriched with all three scores under the 0.005 threshold (LS permutation *p* = 0.00008, KS permutation *p* = 0.00079, Efron-Tibshirani's GSA test *p* < 0.005). Also, when the downregulated transcripts were analyzed for pathway enrichment through the web-based ConsensusPathDB [31], transcripts belonging to the extracellular matrix set emerged as the category more enriched. Indeed, we observed downmodulation of several transcripts belonging to members of the integrin and of the laminin family, with documented interactions among them and in the syndecan family (SDC4 and SDCBP), which can function as both interaction partners for integrins, and for facilitators in the action of membrane ligands such as FGF2 and EGF. We hypothesized that this decrease in proteins involved in cell/extracellular matrix (ECM) interaction might contribute to the proliferation and migration inhibition exerted by miR-135b both by diminishing integrin-mediated signaling and by reducing EGF and FGF2 effects.

## DISCUSSION

Growing evidence has revealed that miRNAs play crucial roles in tumorigenesis, angiogenesis, invasion, and apoptosis in various types of tumor, including GBM [24]. It has been recently demonstrated that miRNA expression profiles are particularly useful for subclassification of GBM. Recent studies have shown that miRNAs could play a role in determining cancer stem cell properties, contribute to treatment resistance, and suggest that miRNAs are not only putative biological markers for diagnosis, but also one of the most promising target for GBM treatment [32]. Anyway, although several studies have tried to identify miRNA profile for GBM, a specific expression signature is not still well-defined due to discrepant results encountered in literature between GBM miRNA expression studies. These discrepancies could mainly be due to the adopted techniques to evaluate their expression, the source of neoplastic specimens and the choice of reference samples used in the analysis. It has been shown that it is more advisable to compare miRNA expression data within similar experimental conditions which, in particular, are strictly dependent on the non-brain neoplastic reference [33]. In line with this observation our results showed that a comparison between cultured cells and tumor tissues should be interpreted very carefully for several reasons including micro environmental factors that could regulate their expression. Thus, in this study, we decided to choose normal neural stem cell lines as control group to analyze miRNA expression in our collection of GSCs.

Here, we showed that miR-135b is downregulated in GSC lines and in commercial GBM cell lines, U87MG and T98G, compared to normal adult and fetal neural stem-like cells. The analysis of miR-135b expression in GBM tissue samples showed a slight but not significant, increase compared to normal brain tissue however, in both tissue samples, miR-135b levels were lower than in cell cultures. The expression of miR-135b in GSCs could be due to different mechanisms including the EGF machinery and microenvironment [34]. Xenograft experiments, indeed, showed that tumor generated by GSC injection expressed lower level of miR-135b than the parental cell line, comparable with GBM samples.

Even though a pro-tumorigenic function has been described for miR-135b in different cellular systems, functional characterization of this miRNA in GSC showed a tumor suppressive behavior. Thus, restoration of miR-135b expression significantly impairs GSC tumorigenic ability *in vitro* and *in vivo*. We identified ADAM12 and confirmed SMAD5 and GSK3β as direct targets of miR-135b. GSK3β has recently been shown to be a crucial enzymatic regulator of a diverse number of cellular functions including cell structure, metabolism, and survival [35]. Although the role of GSK3β in the regulation of apoptosis is controversial, it has been recently demonstrated a role for GSK3β in mediating glioma cell proliferation arrest, decreased clonogenicity and induction of apoptotic cell death through both the extrinsic and intrinsic apoptotic pathways both *in vitro* and *in vivo* [36]. On the other hand, inhibition of GSK3β activity results in c-MYC dependent glioma cell death through multiple mechanisms, all of which converge on the apoptotic pathways. GSK3β may therefore be an important therapeutic target for gliomas [36-38].

One of the most common signaling changes in GBM is the aberrant activation of various receptor tyrosine kinases [39]. In particular, EGFR receptor activation by either overexpression or mutation is a common oncogenic event in more than 50% of GBM [40]. EGFR signaling regulates the proliferation and tumorigenic ability of GSCs by inducing ID3 and ID3-regulated cytokines (GRO1, IL-6, and IL-8), which play a crucial role to make the tumor microenvironment suitable for GSC maintenance. EGFR-mediated ID3 expression is regulated by SMAD5, which is directly phosphorylated by AKT [41]. ADAM12 is a member of the Zn^2+^ dependent metalloproteinase superfamily, and is expressed as both membrane-bound and secreted enzyme. Overexpression of ADAM12 is implicated in the onset and progression of a number of disease conditions, including cancer [42]. Human ADAM12 exists in two forms that arise from alternative splicing; the prototype membrane-anchored protein (ADAM12m) and the shorter secreted type form (ADAM12s) [43]. It has been demonstrated that ADAM12m is selectively expressed in GBM tissues and that its expression levels is directly correlated with MIB1-positive cell index of the malignant astrocytic tumors [27]. This suggests the possible involvement of ADAM12m in the proliferation of GBM that is mainly sustained by enhanced EGF signaling through EGFR. The membrane-bound form of EGF-like ligands has limited biological activity and must be cleaved in a process known as ectodomain shedding to become fully functional [44]. ADAM12 plays an important role in the regulation of ectodomain shedding, resulting in production of soluble growth factors [30]. Heparin-binding EGF-like growth factor (HBEGF), a known EGFR ligand produced as a transmembrane protein needing enzymatic cleavage to be active, is an ADAM12 substrate, and has been implicated in EGFR dysregulation [30]. Moreover, growing evidences have indicated for ADAM12 an important role in supporting tumor cell adhesion, which is mediated through binding of the cysteine-rich domain of ADAM12 to syndecans, cell surface proteoglycans [45, 46]. Because syndecans become expressed in the reactive astrocytes surrounding the region of the brain necrosis, it has been speculated that ADAM12 might facilitate GBM cells to attach to the reactive astrocytes present near the leading invasive edge or, alternatively, it might be involved in mutual cell attachment and spreading of GBM cells to form tumor cell aggregates that are commonly observed in human GBM during the invasive growth [27]. Syndecans and/or β1 integrin are required for ADAM12-mediated cell attachment and spreading [45]. In line with this evidence, we observed that miR-135b expression is coupled to a significant decrease in the expression of SDC4 and β1 integrin transcripts, along with other ligands and receptors of the ECM. ADAM12 has potential to emerge as a successful drug target, although targeting the metalloproteinase domain with any specificity will be difficult to achieve due to structural similarity between the ADAM members and matrix metalloproteinase (MMP) family of enzymes. Moreover, since miRNAs can modulate simultaneous and distinct functions such as tumor growth, invasion, and angiogenesis in GBM, miR-based therapies may prove more effective than current molecular-target therapies, which target single protein-coding genes belonging to more complex oncogenic signaling pathway and achieve modest changes for more global gene expression. Our data on the role of miR-135b in GBM along with recent progress in the delivery of functional miRNA molecules across the blood-brain barrier [47], reinforce the concept that molecular-targeting therapy based on miRNA expression in GSCs has the potential to allow more effective treatment strategies for this incurable type of cancer.

## MATERIALS AND METHODS

### Cell cultures

Packaging cell line, 293T, were maintained in DMEM (Life Technologies Corporation, Carlsbad, CA) supplemented with 10% (v/v) heat-inactivated FBS, 2 mM L-glutamine, 100 U/ml of penicilline and 100 μg/ml of streptomycin (Invitrogen). Glioblastoma stem-like cells were isolated from surgical samples of adult patients who had undergone craniotomy at the Institute of Neurosurgery, Catholic University of Rome, upon patient informed consent and approval by the local ethical committee. Surgical specimens were subjected to mechanical dissociation and the resulting cell suspension was cultured in a serum free medium supplemented with EGF and bFGF as previously described [22]. Cell lines actively proliferating required 3 to 4 weeks to be established. In these conditions, cells grew as clusters (neurospheres) of undifferentiated cells, as indicated by morphology and expression of stem cell markers such as CD133, SOX2, Musashi-1, and nestin. The *in vivo* tumorigenic potential of GBM neurospheres was assayed by intracranial or subcutaneous cell injection in immunocompromised mice. GBM neurospheres were able to generate tumors with the same expression and histological tissue organization as the human parent tumor. GSC lines were validated by Short Tandem Repeat (STR) DNA fingerprinting. Nine highly polymorphic STR loci plus amelogenin (Cell ID^TM^ System, Promega Inc., Madison, WI) were used. Detection of amplified fragments was obtained by ABI PRISM 3100 Genetic Analyzer (Applied Biosystems, Carlsbad, CA, USA). Data analysis was performed by GeneMapper^®^ software, version 4.0 (Biological Bank and Cell Factory, National Institute for Cancer Research, IST, Genoa, Italy). All GSC line profiles were challenged against public databases to confirm authenticity. To assess clonogenicity, viable cells were dispensed at different densities (1, 3 and 10 cells/well) in 96 well plates by cell sorting (FACS Aria, Becton Dickinson). After 10-14 days, the wells with growing clones were enumerated, and results were analysed by the Extreme Limiting Dilution Assay (ELDA) software (http://bioinf.wehi.edu.au/software/elda/) [48]. Data concerning patients and GSCs are summarized in Supplementary Table S1. Human adult neural stem cell line, NS5, were isolated from human neural adult tissue obtained following the ethical guidelines of the NECTAR and the Declaration of Helsinki from patients undergoing particularly invasive neurosurgery as previously described [23]. Human neural progenitor cell (HNPC) lines were purchased by Lonza (Lonza Inc. Walkersville, MD, USA). Normal neural adult and fetal stem-like cell lines were cultured in the same serum free medium supplemented with EGF and bFGF used for GSC lines. The U87MG and T98G cell lines were purchased from ATCC and were cultivated in the recommended media (see www.atcc.org for details). The TB10 cell line was established in our laboratory from a human GBM tumor under conventional adherent culture with serum-containing medium [49].

### Plasmid constructs and lentivirus infection

The miR-135b precursor was cloned in the 3′ untranslated (UTR) region of RFP in the pTRIPZ doxycycline inducible lentiviral vector (Thermo Fisher Scientific, Waltham, MA, USA). Primers used for pri-miRNA-135b amplification were: CGGTCTAGACCATTGTGTGAGGCCTTT (Forward) and CCCGATATCACCCCCCAAATCT (Reverse). miRZip™ anti-sense miR-135b and miRZip™ control (NTC) were purchased by SBI (System Biosciences Inc., Mountain View, CA, USA). For ADAM12 constitutive expression ADAM12 cDNA (NM 003474) was cloned into Tween lentiviral vector [23] by XbaI-Xho restriction enzime. Lentiviral particles were produced by the calcium phosphate transfection protocol in 293T packaging cell line and infection performed as previously described [23]. After infection transduced cells were selected with puromycin and Red Fluorescent Protein (RFP) fluorescence was evaluated by FACSCanto (BD Biosciences, Milan Italy) upon doxycycline induction (Sigma Aldrich Inc., Saint Louis, MO).

### Real-time PCR

Total RNA was extracted from cells using TRIzol reagent (Life Technologies Corporation) and from microdissected paraffin embebbed GBM sections using miRNeasy FFPE kit (QIAGEN s.r.l., Milan Italy) Fifty nanograms of RNA were reverse transcribed with TaqMan MicroRNA Reverse Transcription Kit (Applied Biosystems). Real-time PCR for miR-135b (miRBase ID hsa -miR-135b-5p, v21) was performed using TaqMan^®^ MicroRNA Assays protocol (assay ID 002261 Applied Biosystems). All reactions were run in duplicate. Normalization was performed by using RNU6B primer kit (ID 001093, Applied Biosystems). Relative expression was calculated with relative standard curves for miR-135b and the endogenous control. RT-PCR analysis was performed using an ABI Prism 7900 Sequence Detector (Applied Biosystems).

### *In situ* hybridization

*In situ* detection of miR-135b was performed on formalin fixed paraffin embedded GBM samples using miRCURY LNA microRNA ISH optimization kit (Exigon, Vedbaek Denmark) according to manufacturer's instruction.

### Cell growth, migration and colony formation

For the proliferation assay, TRIPZ and TRIPZ-miR-135b GSCs were plated at density of 2×10^4^/ml in 96 well plates in triplicate. Cell proliferation was monitored by counting the cells and confirmed by using the CellTiter-Blue Viability Assay (Promega). Cell proliferation was also evaluated by Bromo-2′-deoxyuridine (BrdU) incorporation using BrdU Cell proliferation ELISA kit (colorimetric) (abcam, Cambridge, UK) according to manufacturer's instruction.

The motility of transduced GSCs was evaluated by plating in Corning FluoroBlok^TM^ Multiwell Inserts System (Corning Life Sciences, Tewksbury, MA, USA), according to manufacturer's instruction. Briefly, 2-3×10^3^ GSCs were added to the upper chambers in stem cell medium. Growth factors (EGF and bFGF) added to the stem cell medium were used as chemoattractant in the lower wells. The plates were incubated for 48h at 37°C, after which the fluorescent dye calcein acetoxymethylester (calcein AM) was added to the lower chamber for 30 min. The cell viability indicator calcein AM is a non-fluorescent, cell permeant compound that is hydrolyzed by intracellular esterases into the fluorescent anion calcein and can be used to fluorescently label viable cells before microscope observation.

Colony formation ability was evaluated by plating a single cell/well in 96 well plates. After 3-4 weeks, each well was examined and the number of spheres/cell aggregates were counted. All the experiments were performed in stem cell medium in the presence of doxycycline.

### Cell cycle assay and apoptosis

For cell cycle analysis 2×10^5^ GSCs were mechanically dissociated and resuspended in Nicoletti's buffer, containing 0,1% Sodium Citrate, 10mM NaCl, 0,1% Triton X-100, 200mg/mL Propidium Iodide (PI) and 200 mg/mL RNAse A [50]. Following 30 min incubation at room temperature cells were acquired with a FACSCanto flow cytometer (BD Biosciences).

Apoptosis was evaluated using caspase 3/7 activity detection. 2×10^4^/ml in 96 well plates in triplicate and caspase activity was evaluated using Apo-ONE^®^ Homogeneous Caspase-3/7 Assay (Promega) according to manufacturer's instruction.

All the experiments were performed in stem cell medium in the presence of doxycycline.

### Xenograft mouse models

Animal experiments were performed in accordance to relevant institutional and national regulations. 2×10^5^ TRIPZ and TRIPZ-miR-135b GSCs, were intracranially injected into male NOD/SCID mice (*n, 6*; 4-6 weeks of age; CD1 NOD-/SCID mice, Charles Rives, Italy). Before grafting, mice were anesthetized with intraperitoneal injection of diazepam (2 mg/100 g) followed by intramuscular injection of ketamine (4 mg/100 g). The animal skulls were immobilized in a stereotactic head frame and a burr hole was made 2 mm right of the midline and 1 mm anterior to the coronal suture. The tip of a 10-μl Hamilton microsyringe was placed at a depth of 3.5 mm from the dura and the cells were slowly injected. Doxycycline administration in drinking water (200 μg/ml) started the day of injection. After 8 weeks of survival, mice were deeply anesthetized and transcardially perfused with 0.1 M PBS (pH = 7.4), followed by 4% paraformaldehyde in 0.1 M PBS. The brain was removed, stored in 30% sucrose buffer overnight at 4° C and serially cryotomed at 20 μm on the coronal plane. Sections were collected in distilled water, mounted on slides, and cover-slipped with Eukitt. Images were obtained with a Laser Scanning Confocal Microscope (IX81, Olympus Inc, Melville, NY, USA).

The cranio-caudal extension of the brain area invaded by GSCs was assessed on serial coronal sections. Then, histological sections 120 μm apart were digitized; on each image, the brain region containing GSCs was demarcated with the cursor and its area calculated by using a commercially available software. To assess the tumor volume, each area of the infiltrated brain was multiplied for the distance to the consecutive digitized section, starting from the tumor epicentre to the cranial and caudal poles of the tumor, and partial volume values were added. The density of tumor cells was assessed by counting the number of GSCs in 10 non-superimposing high power fields across the grafted striatum. Alternate sections were stained with hematoxylin and eosin (H&E) for morphological analysis.

For intracranial implantation of U87MG GBM cells, male NOD-SCID mice were implanted intracranially with 0.2 × 10^5^ TRIPZ and TRIPZ-miR-135b U87MG cells resuspended in 4 μl of serum-free DMEM. Doxycycline administration in drinking water (200 μg/ml) started the day of injection. After 2 weeks of survival, mice were deeply anesthetized and transcardially perfused with 0.1 M PBS (pH = 7.4), followed by 4% paraformaldehyde in 0.1 M PBS. The brain was removed, stored in 30% sucrose buffer overnight at 4° C and serially cryotomed at 20 μm on the coronal plane. Proliferation and cell death were evaluated by immunostaining with Ki67 (Merck Millipore, Darmstadt, Germany) and terminal deoxynucleotidyl transferase dUTP nick-end labeling (TUNEL) assay, respectively. Images were obtained with a Laser Scanning Confocal Microscope (Flouview FV1000, Olympus Inc.).

Subcutaneous implant of T98G GBM cells was performed by injecting 5×10^5^ TRIPZ and TRIPZ-miR-135b T98G cells mixed with 0.1 ml of cold Matrigel. Doxycycline administration in drinking water (200 μg/ml) started the day of injection. Matrigel implants were removed four weeks after grafting under magnified vision, stored in 30% sucrose buffer overnight at 4° C, and embedded in paraffin and sectioned at 3 μm for Ki67 immunostaining.

### MicroRNA target prediction

TargetScan (http://www.targetscan.org), miRanda (http://www.microrna.org), miRBase (http://www.mirbase.org) and TargetMiner (http://www.isical.ac.in) were used for miR-135b target prediction.

### Western blot analysis

Total protein content was extracted from cells using RIPA buffer (20 mM Tris/HCl pH = 7.2, 200 mM NaCl, 1% NP40) and Protease and Phosphatase Inhibitor Cocktails I and II (Sigma-Aldrich). Samples were resolved in SDS-PAGE gels (NuPage 4-12% bis-tris Gel, Invitrogen). Protein expression was analyzed by standard western blot procedure using anti-ADAM12 (Santa Cruz Biotechnology, Santa Cruz, CA, USA), anti-SMAD5 and anti-GSK3β (Cell Signaling Technology, Danvers, MA, USA). The Anti-β-actin monoclonal antibody was used as loading control (Oncogene Research Products, La Jolla, CA, USA). The quantitation of protein expression was determined after normalization to β-actin, by measuring the optical density of respective band blots using the Quantity One software (Bio-Rad Laboratories, Hercules, CA, USA).

### Reporter assay

293T were transiently co-transfected by Lipofectamine 2000 (Life Technologies Corporation) with 0.8 μg of firefly luciferase reporter plasmid containing wild-type or mutated ADAM12 3′UTRs, 40 pmol of either the hsa-miR135b mimic or control-mimic oligonucleotides (Ambion, Life technologies) and with 50 ng of *Renilla* luciferase *pRL-TK* as normalizer. 48 h post-transfection luciferase activity was quantified by Dual Luciferase Reporter kit (Promega Inc.)

### Gene array

Gene array was performed as previously described [51]. Briefly, total RNA was extracted from TRIPZ and TRIPZ-miR-135b GSC#83 transduced cells. RNA was labeled and hybridized to the Affymetrix GeneChip1.0ST array (Affymetrix, Santa Clara, CA, USA) following the manufacturer's instructions. Hybridization values were normalized by the RMA method.

### Statistical analysis

Statistical analyses was performed by means of either “R”v3.1.2 [52] or GraphPad prism v4.0 (GraphPad Software, La Jolla CA, USA, www.graphpad.com). Statistical significance reported on the plots is the following: single asterisks for *p* < 0.05, two asterisks for *p* < 0.01 and three asterisks for *p* < 0.001.

## SUPPLEMENTARY MATERIAL TABLE AND FIGURES


