# Supplementary material for: miR-135b suppresses tumorigenesis in glioblastoma stem-like cells impairing proliferation, migration and self-renewal
Source: Oncotarget. 2015 Sep 30;6(35):37241–56. doi: 10.18632/oncotarget.5925 (PMC4741927; doi:10.18632/oncotarget.5925)
Supplement: Supplementary file 1 [file oncotarget-06-37241-s001.pdf]

miR-135b suppresses tumorigenesis in glioblastoma stem-like cells impairing proliferation, migration and self-renewal

Supplementary Material

Supplementary Table 1. Patient and GSC line characteristics

	Patients										Cell lines	
GSC #	Age (yr)	Sex	KPS (score)	Sympt. (mo.)	Prim Rec	Location	Surgery (type)	Ki 67 (%)	PFS (mo.)	OS (mo.)	CD133 (%)	Estimate Stem cell frequency
1	40	M	80	2.5	P	Temporal	P	20	6.0	12.5	95.8	4.32
23C/23P	77	M	80	2.0	P	Parietal	C	50	1.0	2.0	14.2	3.98
28	72	M	90	1.5	P	Frontal	C	5	6.0	11.5	1.4	5.46
30P/30PT	44	M	80	1.0	P	Frontal	C	10	5.0	7.5	0.2	6.26
61	59	M	80	2.0	P	Occipital	C	35	3.0	6.0	2.5	1.63
62	64	M	80	36.0	R	Frontal	C	10	10.0	14.0	82.7	9.27
67	48	M	60	2.5	P	Parietal	C	20	1.0	2.0	0.2	5.55
68	58	M	70	3.0	P	Parietal	C	10	4.0	10.5	1.2	32.15
70	67	F	80	2.5	P	Parietal	C	20	6.0	9.0	17.3	17.62
74	70	F	60	1.5	P	Frontal	P	15	2.0	8.0	1.5	1.36
76	48	F	90	2.5	P	Frontal	C	15	11.0	16.0	1.8	7.16
83/83.2	52	M	70	0.5	P	Temporal	C	40	3.0	8.0	1.0	2.66
112	49	F	70	2.5	P	Parietal	C	18	3.0	6.0	61.5	4.23
120	53	M	80	25.0	R	Parietal	C	30	2.0	8.0	83.8	31.91
144P	57	M	70	2.0	P	Temporal	C	40	11.5	19.0	13.2	7.45
147	69	F	70	2.0	P	Frontal	C	25	1.0	11.0	0.8	23.65
148	55	M	80	6.0	R	Parietal	C	70	1.0	8.0	94.8	14.64
151	69	M	70	4.0	P	Occipital	C	30	60.0	69.0	0.3	4.21
163	56	M	50	5.0	P	Parietal	C	12	1.0	2.0	1.2	2.33
166	47	M	70	2.0	P	Temporal	P	35	34.0	42.0	10.5	7.26
169	61	M	60	2.5	P	Temporal	C	40	4.0	9.0	6.6	5.21
171	74	M	60	13.0	R	Frontal	P	10	2.0	17.0	3.3	49.73
172	77	F	50	0.5	P	Parietal	C	20	3.0	6.5	2.7	17.95
204	80	F	50	3.0	P	Parietal	C	25	2.0	5.5	84.9	8.13
209	43	F	70	2.5	P	Occipital	P	40	18.0	26.0	0.3	5.42
210	53	M	90	4.0	P	Parietal	C	40	6.5	10.5	95.9	75.77
220	63	M	70	2.0	P	Frontal	P	30	1.5	2.0	16.8	3.9
221	78	M	70	0.5	P	Temporal	P	40	1.0	2.0	64.3	11.06
242	64	M	70	0.5	P	Temporal	P	25	13.0	23.0	0.2	3.30
257	57	M	70	1.0	P	Multicentric	P	50	5.0	19.0	6.2	13.51

KPS, Karnofsky Performance Status; Sympt., symptom duration; Prim., primary tumor; Rec., recurrent tumor; PFS, progression-free survival; C, complete surgery; P, partial surgery; OS, overall survival.

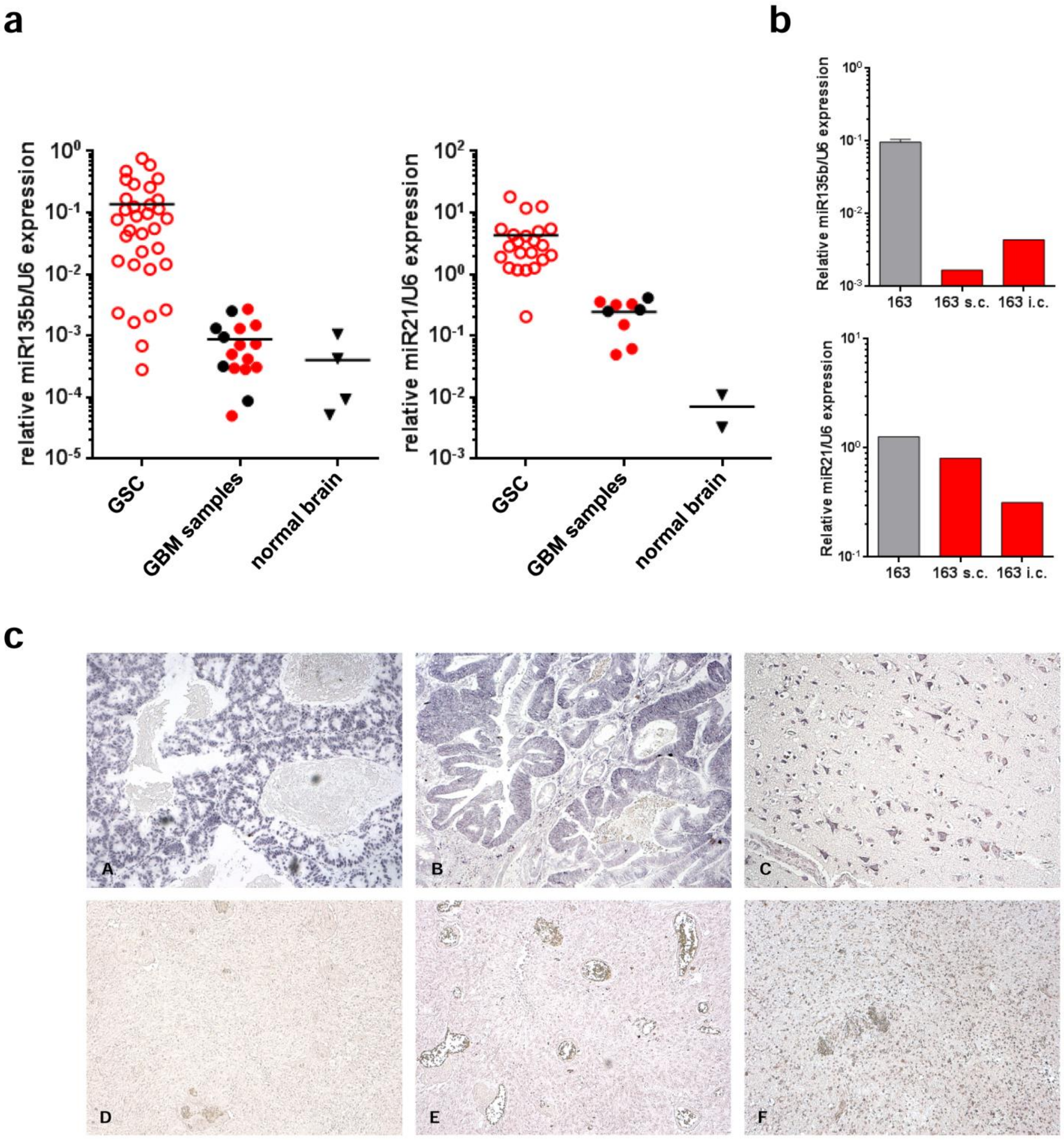

Supplementary Figure 1. a) Expression levels of miR-135b (left) or mir-21 (right) in GBM stem cell lines cultures (GSC), in GBM samples and normal brain tissues. GBM samples from which a GSC cell line were generated are indicated in red. Values are expressed as relative to normal neural stem cells. **b)** Comparison of miR-135b or miR-21 expression levels in GSC culture and tumor xenografts generated by injection of the same GSC line by subcutaneous (s.c.) or intracerebral (i.c.) route. Values are expressed as relative to normal neural stem cells. **c)** Representative ISH for miR-135b (A,C,D,E,F) and U6 control (B) in paraffin embedded sections of colorectal adenocarcinoma (A, B), normal brain (C) and GBM patients (D, E, F). Magnification 100X (A, B, D, E, F), 200X (C).

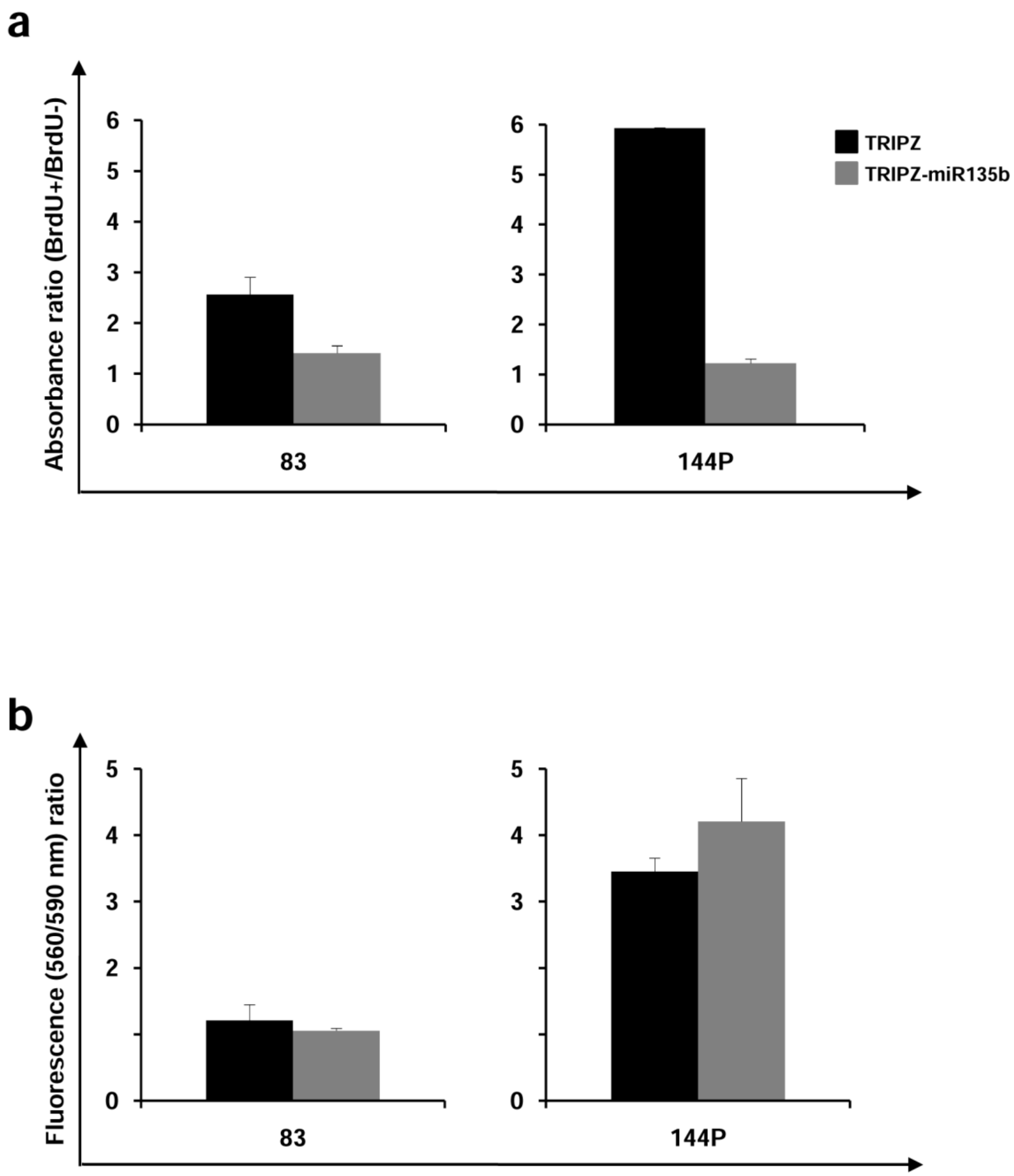

Supplementary Figure 2. a) BrdU incorporation after 24 h pulse in TRIPZ and TRIPZ-miR135b transduced GSC lines #83 and #144P. Absorbance (450-550 nm) has been shown as ratio between BrdU+ and BrdU- wells. Values are reported as mean ± SD from two independent experiments in triplicate. **b)** Caspase activity detected 1 h after incubation with Apo-ONE® Caspase-3/7 Assay Reagent. Fluorescence (560/590 nm) has been shown as ratio with negative control (medium without cells) wells. Values are reported as mean ± SD from two independent experiments in triplicate. All experiments have been performed 6 days after doxycycline induction.

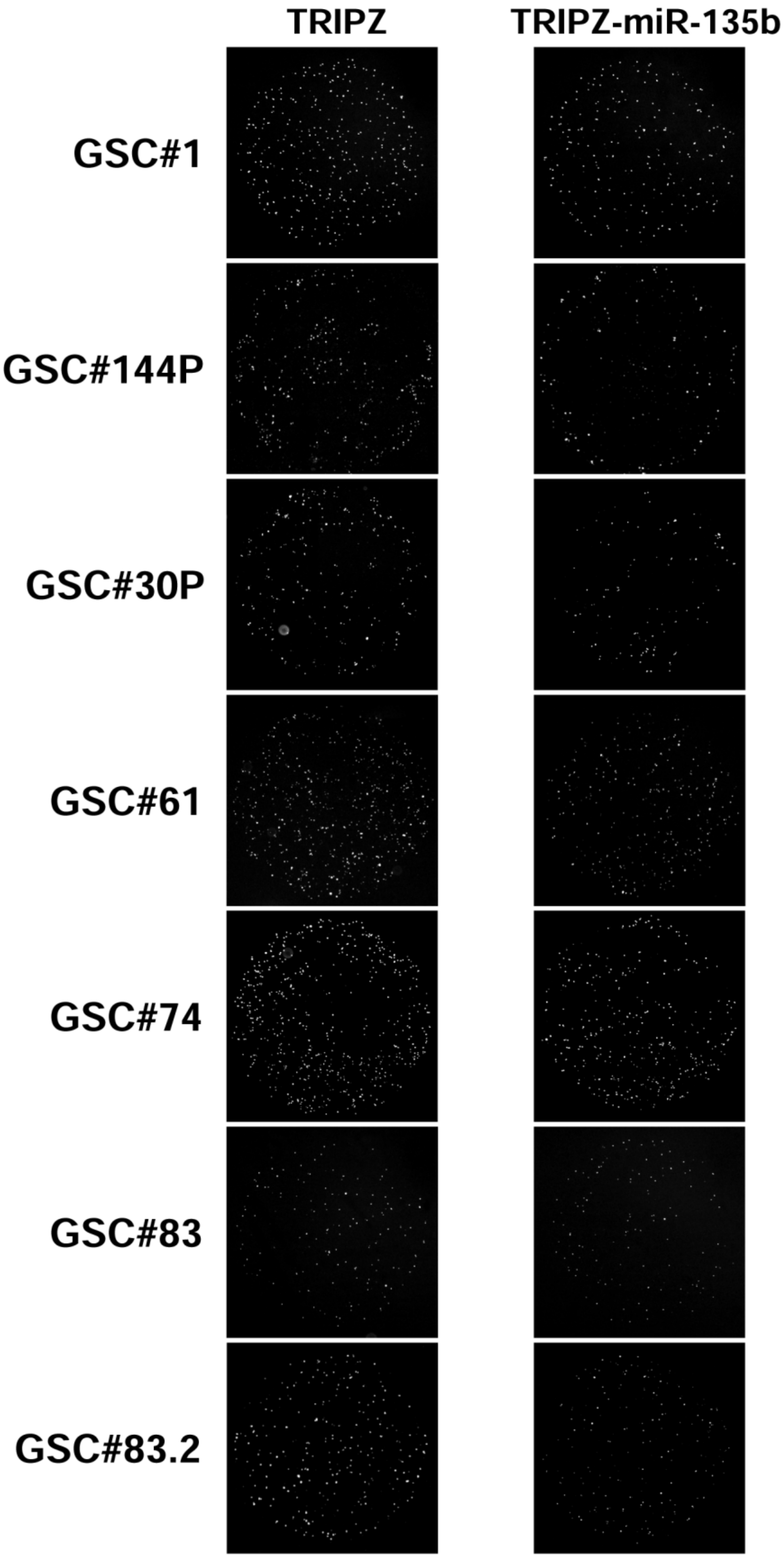

Supplementary Figure 3. Migration assays in GSCs transduced with TRIPZ or TRIPZ-miR-135b vectors, 48h post-seeding.

a

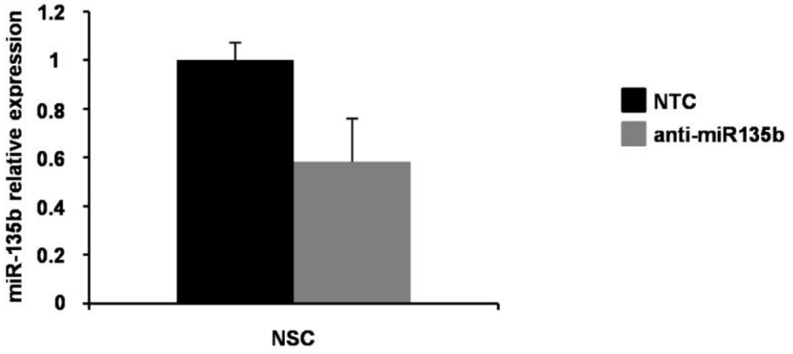

b

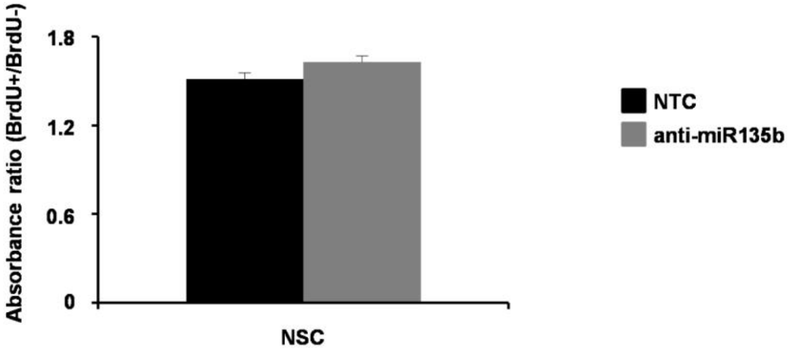

c

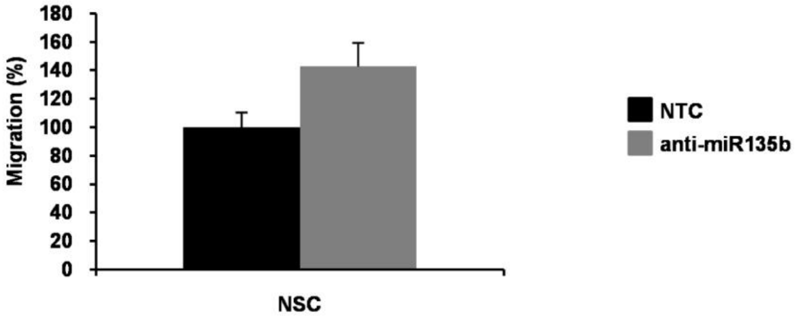

Supplementary Figure 4. a) Expression of miR-135b analyzed by real-time PCR in NTC and anti-miR135b transduced NSC line. **b)** BrdU incorporation after 48 h pulse in NTC and anti-miR135b transduced NSC line. Absorbance (450-550 nm) has been shown as ratio between BrdU+ and BrdU- wells. Values are reported as mean ± SD from two independent experiments in triplicate. **c)** Analysis of migration efficiency in NTC and anti-miR135b transduced NSC line 48h after seeding. Percent values are reported as mean ± SD from two independent experiments in duplicate.

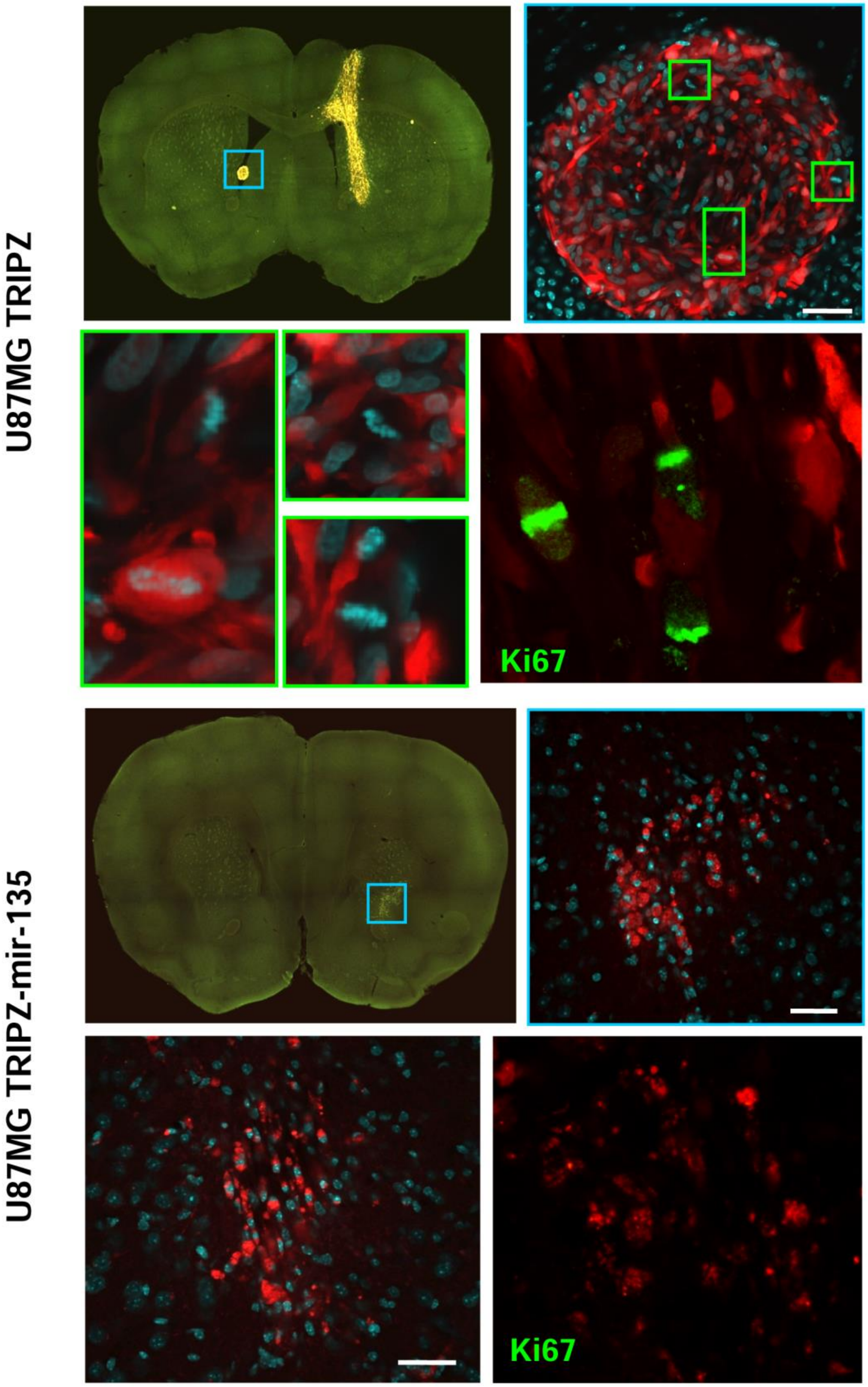

Supplementary Figure 5. Fluorescence microscopy images and Ki67 immunofluorescence of mouse brain specimens at twelve days after injection of TRIPZ or TRIPZ-miR-135b-transduced U87MG (scale bars, 250 µm).

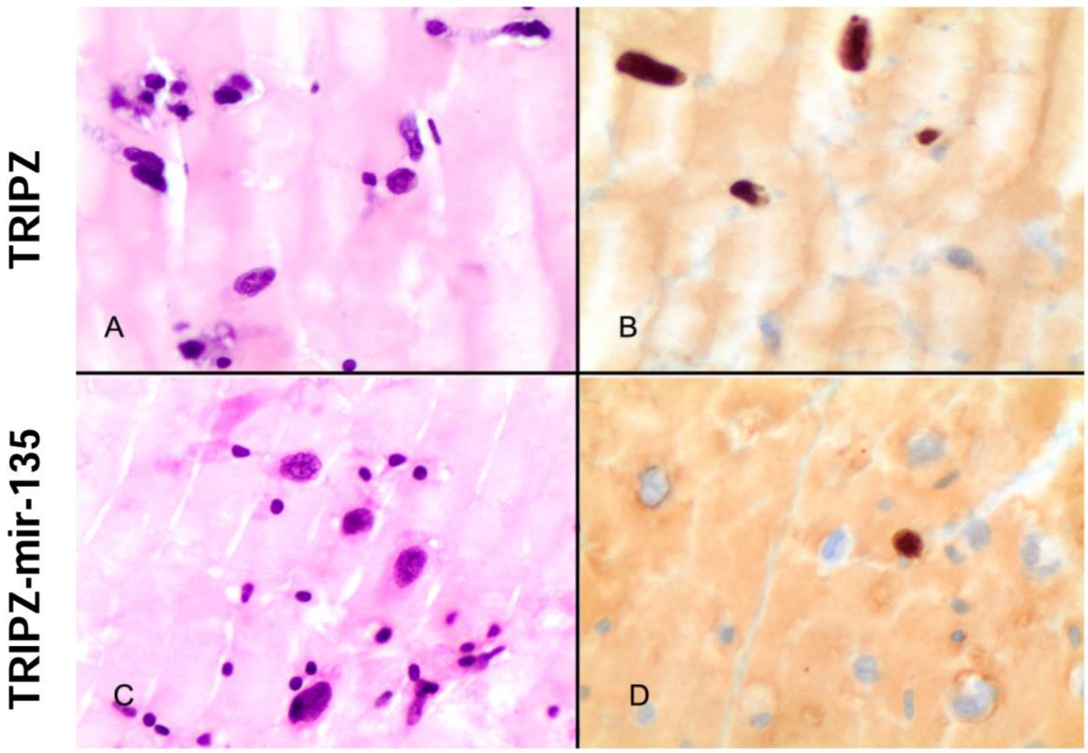

Supplementary Figure 6. Hematoxylin-eosin staining and Ki67 immunostaining of TRIPZ (A and B) or TRIPZ-miR-135b (C and D) transduced T98G Matrigel subcutaneous implants four weeks after grafting.

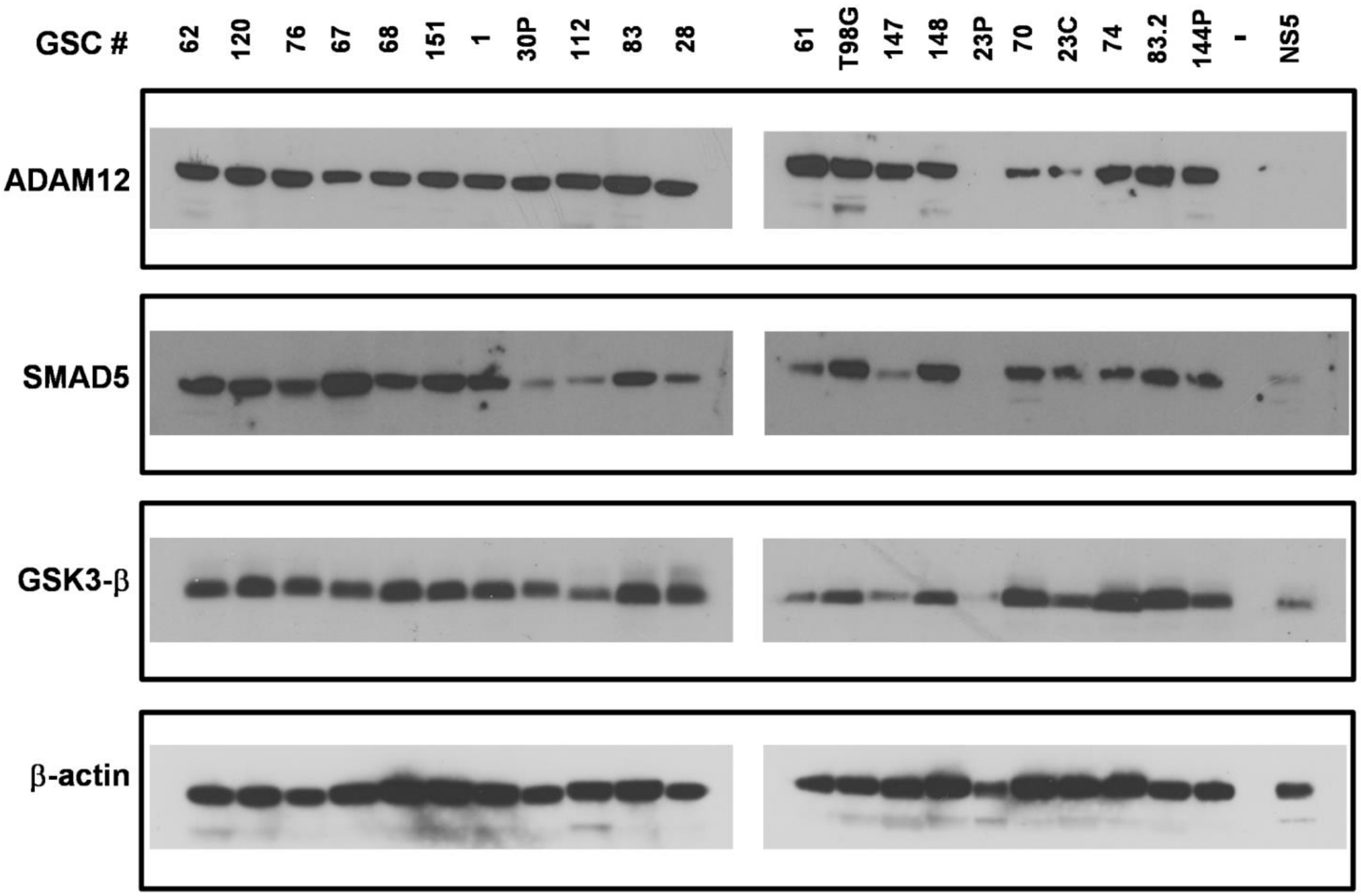

Supplementary Figure 7. One out of two representative immunoblots for ADAM12, SMAD5 and GSK3β in GSCs, T98G GBM and NS5 normal neural stem cell line. β-actin was used as loading control.

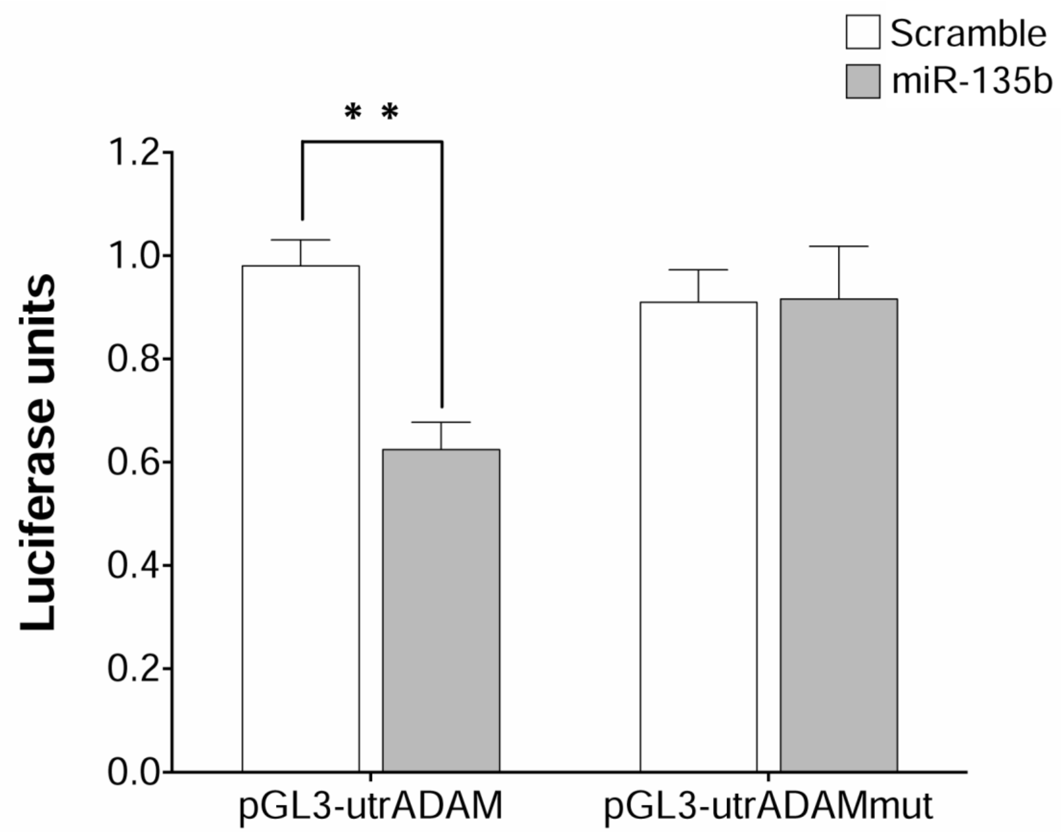

Supplementary Figure 8. Reporter assay of wild-type and mutated target constructs. Histograms show normalized mean values of the relative luciferase activity. Reporter activity is normalized to control plasmid. Data are expressed as means ± SD of three independent experiments.

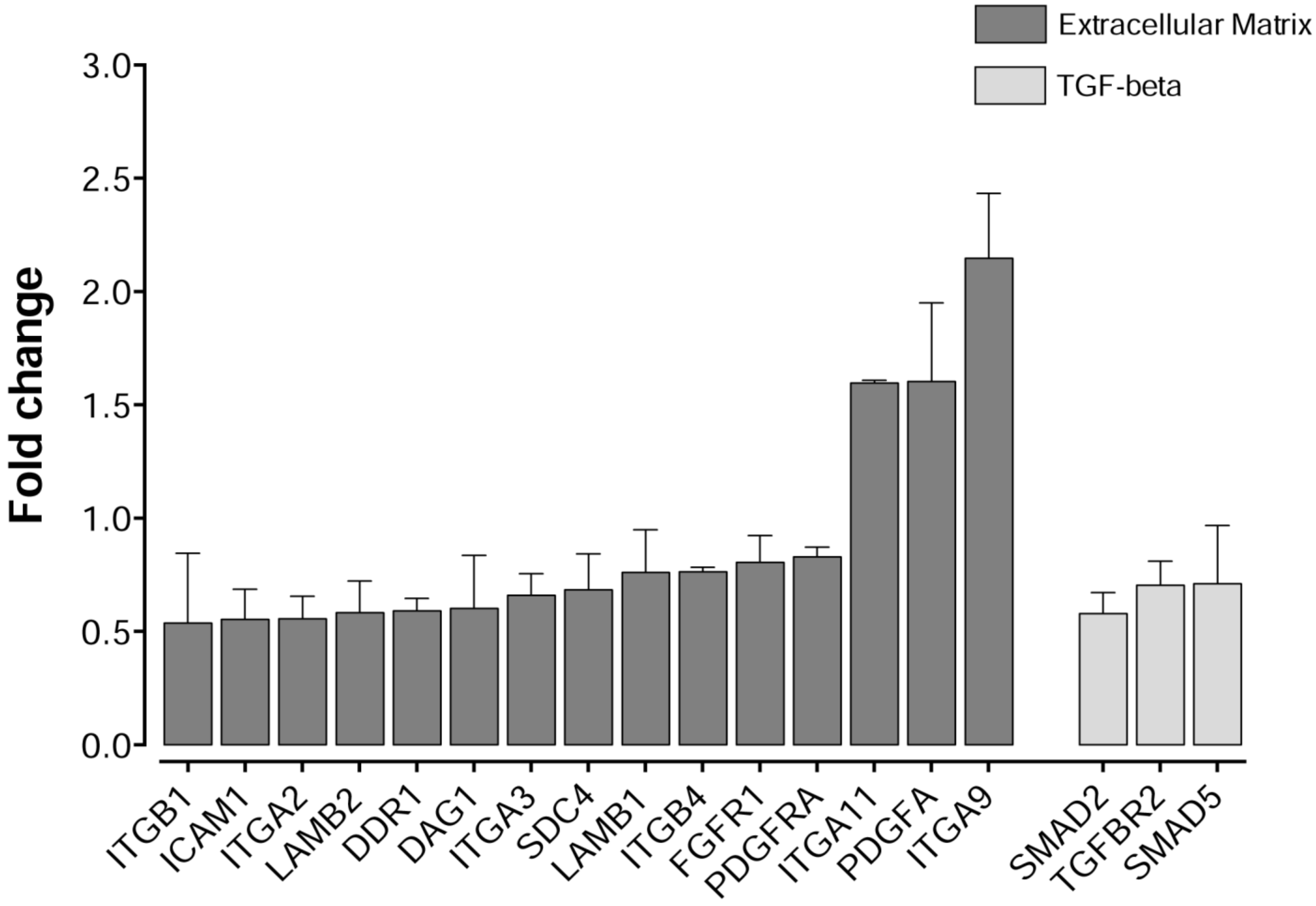

Supplementary Figure 9. Fold change (relative to TRIPZ vector) of transcripts significantly modulated by miR-135b are reported as means ± SD from two different experiments on GSC line #83.
